# An efficient binary salp swarm algorithm for user selection in multiuser MIMO antenna systems

**DOI:** 10.1038/s41598-025-00772-2

**Published:** 2025-05-12

**Authors:** A. Sasikumar, Logesh Ravi, Malathi Devarajan, Abdulaziz S. Almazyad, Shuvodeep De, Guojiang Xiong, Seyed Jalaleddin Mousavirad, Ali Wagdy Mohamed

**Affiliations:** 1 Department of Data Science and Business Systems, Faculty of Engineering and Technology, SRM Institute of Science and Technology, Kattankulathur, Tamil Nadu 603203 India; 2 Centre for Advanced Data Science, Chennai, Tamil Nadu 600127 India; 3 School of Electronics Engineering, Vellore Institute of Technology, Chennai, Tamil Nadu 600127 India; 4 School of Computer Science and Engineering, Vellore Institute of Technology, Chennai, 600127 India; 5 Department of Computer Engineering, College of Computer and Information Sciences, King Saud University, 11543 Riyadh, Saudi Arabia; 6 Virginia Tech, Blacksburg, USA; 7 Guizhou Key Laboratory of Intelligent Technology in Power System, College of Electrical Engineering, Guizhou University, Guiyang, 550025 China; 8 Department of Computer and Electrical Engineering, Mid Sweden University, Sundsvall, Sweden; 9 Operations Research Department, Faculty of Graduate Studies for Statistical Research, Cairo University, Giza, 12613 Egypt; 10 Applied Science Research Center, Applied Science Private University, Amman, 11931 Jordan; 11 Centre for Research Impact and Outcome, Chitkara University Institute of Engineering and Technology, Chitkara University, Rajpura, Punjab 140401 India

**Keywords:** Multiuser MIMO, User scheduling, Binary salp swarm algorithm, Metaheuristics optimization, Antenna, Electrical and electronic engineering, Computational science

## Abstract

The past ten years have seen notable research activity and significant advancements in multiuser multiple-input multiple-output (MU-MIMO) antennas. An MU-MIMO antenna system must accommodate many subscribers without additional bandwidth or energy. User scheduling becomes a critical strategy to take advantage of multiuser heterogeneity and acquire maximum gain in systems where the total number of recipients exceeds the number of transmitting antennas. Due to their high computational cost, many user selection methods currently in use, such as greedy algorithms and exhaustive search are unsuitable for MU-MIMO systems. A suitable scheduling mechanism is essential for the various users in an MU-MIMO system to utilise bandwidth and enhance the system’s total rate effectively. In this article, we proposed a user and antenna scheduling with a population-based meta-heuristic approach, namely the binary salp swarm algorithm (binary SSA), to increase the system sum rate with low computing complexity. We specifically used a population-based meta-heuristics optimisation technique to simulate the user scheduling problem in MU-MIMO systems, characterising complicated issues with binary decisions. Additionally, binary SSA significantly outperforms existing population-based models, such as the binary bat algorithm (binary BA), PSO, SSA, FPA and binary flower pollination algorithm (binary FPA), regarding system throughput/sum rate. The proposed binary SSA technique also effectively achieves a system sum rate compared to a random search scheme and other existing suboptimal scheduling methods. Compared to binary BA and binary FPA approaches, the binary SSA has a higher convergence rate and superior searching capabilities. The simulation outcomes show the proposed binary SSA-based scheduling scheme delivers noticeable performance benefits.

## Introduction

Wireless technology is widely used in many industries, notably telecommunications, aviation, medicine, and the armed forces. These systems are being used more frequently, prompting manufacturers to concentrate on improving wireless gadgets. As a result, cognitive technology has advanced significantly in recent years^1,2^. The wireless technology and antenna innovations were made possible after the considerable advancements in circuits and mathematical data processing approaches. Electromagnetic fields connect these devices with cell phones, computers, ground stations, and other equipment^3^. One of the most crucial components of wireless networks is the antenna. These components convert electrical energy into electromagnetic waves, then broadcast into space, and vice versa. Since the antenna occupies the most space in the transmission chain, expanding its overall size makes installing a wireless device challenging in a restricted space^4^. One of the primary objectives of antenna manufacturers in recent years has been to reduce the dimensions of antennas. Tiny antennas are utilised to produce wireless equipment using micro-fabrication techniques^5,6^. In reality, the length of an average antenna that functions at a given frequency is often on the order of a half-wavelength of that wavelength. For instance, the typical length of an antenna that resonates at 1 GHz with a dielectric constant 2.2 is roughly 100 mm. For many devices, this length needs to be fixed. Additionally, most devices like satellites, RFID chips, and phones require several antennas^7,8^. Therefore, creating tiny antennas will be a problem for researchers as wireless technology advances.

For the successful administration of network assets, emerging wireless systems must have an essential and precise knowledge of design concepts and control methods. The cores of wireless transmission systems are resource allocation policies, which attempt to ensure the requisite QoS in the end point while assuring performance and optimised network to maximise operators’ income^9^. A wide range of network operations, consisting of scheduling, rate of transmission control, control of power, capacity reserve, call admittance oversight, transmitters task, and transfer, may be included in the distribution of resources administration for wireless networks. Generally, a resource allocation rule is characterised by the following elements:

i) A multiple accessibility method and scheduling element which divides resources between user’s requirement to individual QoS specifications.
ii) A signalling strategy will permit continues delivery of separate streams of data to the scheduled users
iii) Rate allocation and power management which ensure QoS and manage interest disturbance.

The many types of multiple access techniques include conventional and non-orthogonal ones. The previous is a traditional approach that allows one user one broadcast interval’s worth of radio assets, such as a code, sub-carrier, or time slot. Since co-resource interference is not a problem, the dependability of orthogonal multiple access systems is their key feature. Numerous performance indicators, including throughput, equality, and QoS, can be optimised with manageable complexity by the resource allocation rule^10^. The quantity of system-available radio assets, or the multiplexing gain, which refers to the number of scheduled users, is constrained. Several users simultaneously overlay their signals over the same radio resource in non-orthogonal multiple access, with an opportunity for interfering with one another. By implementing signal handling and transmission methods at the broadcaster and recipient sides in this design, co-resource disturbance can be reduced. Its challenge is to meet the high data rate requirements and system effectiveness anticipated in generating wireless systems; such strategies use several resource fields, such as power, software, or spatial domains.

Over the past few years, MU-MIMO architecture has been thoroughly researched from the theory and practice perspective. Massive MIMO or large-scale MIMO is a current development of MU-MIMO system, uses a few hundred antennas at the base station to transmit several data streams concurrently to several users. One of the potential wireless solutions to satisfy the enormous bandwidth need needed by 5G wireless networks has been highlighted as massive MIMO^11^. The geographical position of the receivers in MU-MIMO situations is unpredictable, making joint detection impossible, which makes the downlink broadcast particularly difficult. The main motive is to develop dynamic data transmission techniques to a group of connected users to take advantage of the spatial multiplexing gain provided by MU-MIMO systems. Establishing such a user set, nevertheless, is a difficult issue involving all aspects of resource strategy allocation, such as the transmitting device’s power management tactics, signalling schemes, rate allocation, and unique QoS demands. A wide range of powerful signal processing techniques can improve the efficiency of MIMO networks. These resources come in the form of signal spaces, transmitting powers, time frames, sub-carriers, codes, and users, to name a few. A trade-off between optimal results and practicality is implied by efficient allocation strategies over a broad range of resources. Achieving optimality may take a lot of work by solving optimisation issues over a set of constant and continuous variables. The scalability suggests that inefficient resource distribution occurs through relaxing and reorganising optimisation issues whose solutions can be identified via useful and trustworthy methods.

In summary, this research aims to introduce an innovative and effective method based on the binary salp swarm algorithm to optimize user scheduling strategies in multiuser MU-MIMO antenna systems. To meet the increasing demands of contemporary 5G wireless network applications, the results of this study may pave the way for improved spectral efficiency, throughput, and user fairness in wireless communication networks.

### Contribution

The existing literature on MIMO transmission is extensive, and this work adds to it by classifying the various components of wireless networks and energy distribution plans. Users with separate channels offer fresh diversity to improve overall performance. However, in MU-MIMO structures, the increased variety is realized when multiple people share the same spectrum continuously, unlike networks where every user receives a specialized (orthogonal) channel^12,13^. When several antennas are considered at both sides of the network, separate signals can be spatially guided using precoding techniques. This leads to the conjunction of multiple information being transmitted to neighbouring users. The examination and categorization of linear and non-linear precoding strategies, considering the quantity of stream data accessible at the sender. The network scenario and the antenna conditions are some of the primary contributions. Each precoding technique relies on separate MU-MIMO channel properties to properly utilize the spatial domain. The related work thoroughly describes measures that measure spatial reliability, which may be applied to user selection and precoding speed improvement.

The spectrum efficiency, rate of error, fairness, and QoS are typical metrics used in the MU-MIMO domain to evaluate quality^14^. To build reliable resource allocation algorithms, the proposed model needs to satisfy the cross-layer model, precoding method, antenna setup, and upper-layer network. The discussion and categorization of several optimization criteria and the overall limitations for MU-MIMO systems are additional requirement of user scheduling. The proposed classification considers the availability of upper layer needs, transmitter channel information, and antenna layout. Early studies on MU-MIMO have noted that monitoring the immediate channel variations for scenarios with a single transmitter can strategically improve resource allocation. However, many methods have been created recently for extremely varied and diverse MIMO circumstances^15^. In the paper, cutting-edge scheduling techniques for MU-MIMO systems with a single and multiple transmitters are categorized.

The main aim of this article is not to design a downlink antenna but rather to concentrate on a basis scheduling model to achieve better efficiency. Our main objective is to implement optimal scheduling model for MU-MIMO, describing the real-time issues and creating an objective function to schedule efficient resource allocation. The major development of this work is listed as follows:

1. A binary model-based scheduling technique is proposed in this article. We implemented MU-MIMO networks with a feedback model to reduce SINRs related to each user in the system.
2. The swarm intelligence model is described concerning the MU-MIMO system to schedule the users effectively.
3. Compared to another basic scheduling method, the proposed binary salp swarm algorithm does not require any constraints mechanism for feedback overhead.
4. The simulation-based swarm intelligence model is implemented for MU-MIMO downlink systems.

The article is structured as follows. In “Related work” section, the basic theory of MU-MIMO-based existing scheduling models and their challenges are discussed. In “MU-MIMO system description” section, we provided the basic model of the MU-MIMO network for various users of the downlink system. “Scheduling using binary salp swarm algorithm” section is presented the proposed binary salp swarm algorithm and its objective function for the user scheduling model. “DPC scheduling using binary SSA” and “Binary FPA for user scheduling” sections provides the detailed steps of user scheduling and DPC model implementation. In “Experimental results and discussions” and “Complexity analysis” sections we implemented the simulation of binary swarm intelligence-based MIMO scheduling, and its performance compared with existing methods. “Convergence analysis” section provides the conclusion of MIMO scheduling using swarm intelligence techniques.

## Related work

Service providers are drawn to multiple-output (MIMO) systems because they can deliver excellent throughput without requiring additional transmitting power or capacity. The basic goal of MIMO is to boost system throughput and dependability by taking advantage of the varied signals connected with different communication antennas. It has been noted that the MIMO broadcasting channel (BC) has M and N number of antenna. In that M is the number of antennas used for transmission, and N is the number of reception antennas. MIMO has a channel bandwidth that is t time more than single-input single-output (SISO) wireless networks^16^. Additionally, MIMO systems provide spatial diversity, multiplexing, improved array gain, and reduced noise. The antenna and client scheduling may be used to improve the system sum rate for multi-user MIMO (MU-MIMO) systems. In many ways, MIMO systems are superior to SISO systems.

Researchers have recently emphasized using multiple number of antennas in the base station. Massive MIMO (mMIMO) is the term used to refer to these multi-antenna networks. The safety of the mMIMO wireless transmission technology is an issue. The eavesdropper could decode the signals the sender sends if the sender channel is better than the receiver antenna. As Shen et al.^17^ detailed, mathematical model utilizing generated noise are deployed to reduce eavesdropping quality. Another approach to ensuring consistent transmissions is the deployment of a smart jammer, which interacts with user-end message reception while ensuring desirable energy efficiency. Additionally, Shen et al. minimize the cost of mMIMO transmission while guaranteeing continuous connection. Antenna connections with high levels of mobility face difficulties as well^18^. In Xin et al., an adaptive MIMO transfer technique, that can improve the antenna performance in the dynamic environment have been implemented in response to these issues and analyses of channels’ spatial and temporal diversity^19^. The large increase in traffic is another issue that mMIMO faces. Because of this, improving network capacity is crucial.

Additionally, the BS and users’ power usage must be decreased. The researchers tackled these problems in earlier investigations. Power-controlled optimisation for MIMO downlink networks has been studied by Zhou et al.^20^. by combining transmission and energy resource management approach optimisation. A joint antenna choosing and power management algorithm is proposed using various techniques, including the Lyapunov optimisation strategy, the bisection method, and nonlinear fractional computing. A joint user and antenna scheduling approaches for circulated mMIMO with constrained downlink ability were presented by the author Xu et al.^21^. These plans maximise the network’s sum-rate capability.

Using dirty paper coding (DPC), which concurrently schedules numerous users, MU-MIMO BC can reach a throughput close to ideal^22^. The extensive search algorithm (ESA) is used for DPC. The MIMO network sum rate for DPC is demonstrated. The user information is encoded and decoded in reverse using the DPC method. Because each user’s encoding is distinct, any interference brought on by already encoded users is already cancelled. When K >  > M, customers are being serviced by the multi-antenna BS, the zero-forcing beamforming pre-coding method, which is simpler, achieves the best results. However, since there are no restrictions in end terminals users use that can be encoded continuously. The DPC needs to be more sophisticated to be deployed realistically, owing to hardware constraints.

The user interfaces’ coding pattern is crucial to the DPC method. DPC is a revolutionary transmission method that allows data to be efficiently sent from BS to many users simultaneously. The ideal value of the utility function can be calculated once the task of looking over all potential user pairings has been completed. As stated in (1), there are a total number of arranged selections:

1 $$N_{OrderedUsers} = \sum\limits_{k = 1}^{M} {(K!)} \left( {\begin{array}{*{20}c} K \\ k \\ \end{array} } \right)$$

Foschini looked into the ability provided by multi-element arrays and the scenario of routes connecting the antenna components subject to Rayleigh fading^23^. Longfei et al. evaluated the benefits of rate-splitting multiple access downlink to enhance the network performance of antenna than a rayleigh fading while including various rate-allocation techniques^24^. Yuhao et al. provided a joint fair resource allocation techniques for user selection, energy optimization and modulation. The proposed method generalises the use of single cell downlink mMIMO antenna which consists of sequence number of time slots over system power constraints. Authors developed antenna transmission using regularized zero-forcing techniques to enhance the modulation accuracy^25^. Ajibi et al.^26^ reviewed different several scheduling techniques for MIMO system networks. According to a MU scheduling strategy put forth by Ajibi et al., the M transmitter antenna separately sends signals to the most advantageous M users according to an excellent SINR. The proposed scheduling methods have been suggested using SINR and without considering the transmission channel side data.

In practice, DPC typically uses a large search space. Methods to restrict the search space for effective seeking capabilities have been described by Pattanayak et al.^27^. Additionally, it could be more computationally efficient because DPC involves many complex adds and multiplications and requires much more time to compute. This goes beyond the technique’s brief periods of coherence. As a result, many soft computing methods have been applied in the past to various MU multiple-antenna network designs. The naturally inspired optimization techniqueis tested for the current MU-MIMO system model to quickly get the best outcome in recent times. We were confident to evaluate SSA effectiveness for the current network architecture by several prior studies of SSA in other disciplines^28^. Particle swarm optimisation (PSO), ant colony optimisation (ACO), bacterial foraging optimisation (BFO), flower pollination algorithm (FPA), and other swarm intelligence-inspired optimisation algorithms were all demonstrated in these studies to be comparable with or like SMO in terms of dependability, effectiveness, and precision. SMO works better in limited optimisation tasks, as demonstrated in^28^. Different restrictions must be satisfied for the current optimisation problem to be considered in this study. To achieve the highest system efficiency with less time difficulty than DPC (i.e., with a lower computational cost), all these investigations have persuaded us to implement the logical variant of the nature inspired SSA technique for the MIMO communication scheduling issue. These earlier investigations have demonstrated that SSA is incredibly effective and efficient in resolving various optimisation issues connected to various engineering areas^29^. It has been demonstrated in this study that the binary SSA has successfully achieved close to ideal system throughput. This can be described by pointing out that binary SSA finds the optimal user and channel set configuration for the MU-MIMO system, resulting in a system throughput close to the theoretical limit (DPC).

Additionally, it has been demonstrated that binary SSA has significantly lower computational and time costs than DPC (ESA). In addition, the suggested binary SSA’s time requirement falls well inside a coherence time frame. Section 4 presents a comprehensive analysis of the suggested binary SSA. The genetic algorithm (GA) is a soft computing algorithm inspired by nature. GA has been employed in the literature for several scheduling issues for single and multi-carrier MIMO systems^30^. This study compares the suggested binary SSA algorithm with various system performance metrics considering FPA and GA. This research presents stability analysis for various optimization techniques. This work adapts binary SSA for MU-MIMO downlink networks to increase the sum rate. Compared to the ESA (DPC), the computation cost is lower here, with less feedback scheduling. Different simulation outcomes are used to show the efficiency study of binary SSA. Binary SSA’s effectiveness study is contrasted with that of DPC, random search, inefficient scheduling algorithms from earlier research, GA, and binary FPA. Furthermore, this research has demonstrated that the suggested binary SSA outperforms the GA, binary FPA, and binary BA regarding system performance and sum rate. When used to solve complicated multiuser MIMO antenna selection issues, several of the current metaheuristic algorithms including GA, PSO, and the traditional SSA have delayed convergence. Because of their ineffective exploration and exploitation balance, these algorithms frequently need many iterations to arrive at a nearly optimum answer. One of the biggest problems with traditional user selection techniques is their computational complexity.

Although exhaustive search methods provide optimality, their exponential complexity makes them unfeasible. However, even while metaheuristic-based approaches are more effective than brute-force approaches, they frequently need a large amount of processing power: example GA and PSO: These techniques use population-based assessments, which raises the computing costs of each iteration. Furthermore, GA necessitates extra processing costs for activities involving crossover, mutation, and selection. Although metaheuristic algorithms are frequently employed for user selection, the optimization efficiency of these algorithms varies greatly depending on the issue complexity, initialization techniques, and algorithm parameters. The premature convergence, because there is insufficient variety in the search process, many traditional approaches, such as PSO and FPA, experience premature convergence, in which the algorithm becomes stuck in local optima. Algorithms such as FPA and GA may struggle to balance exploitation and exploration. Slow convergence or less-than-ideal solutions may result from this mismatch. To meet these difficulties, a more effective binary optimization technique that increases convergence speed lowers computational overhead and improves solution quality. Combining enhanced mechanisms for exploration–exploitation balance, adaptive learning strategies, and practical encoding approaches designed for binary optimization in MIMO user selection, the Binary Salp Swarm Algorithm (BSSA) presented in this study seeks to improve the conventional SSA.

## MU-MIMO system description

**Fig. 1:**
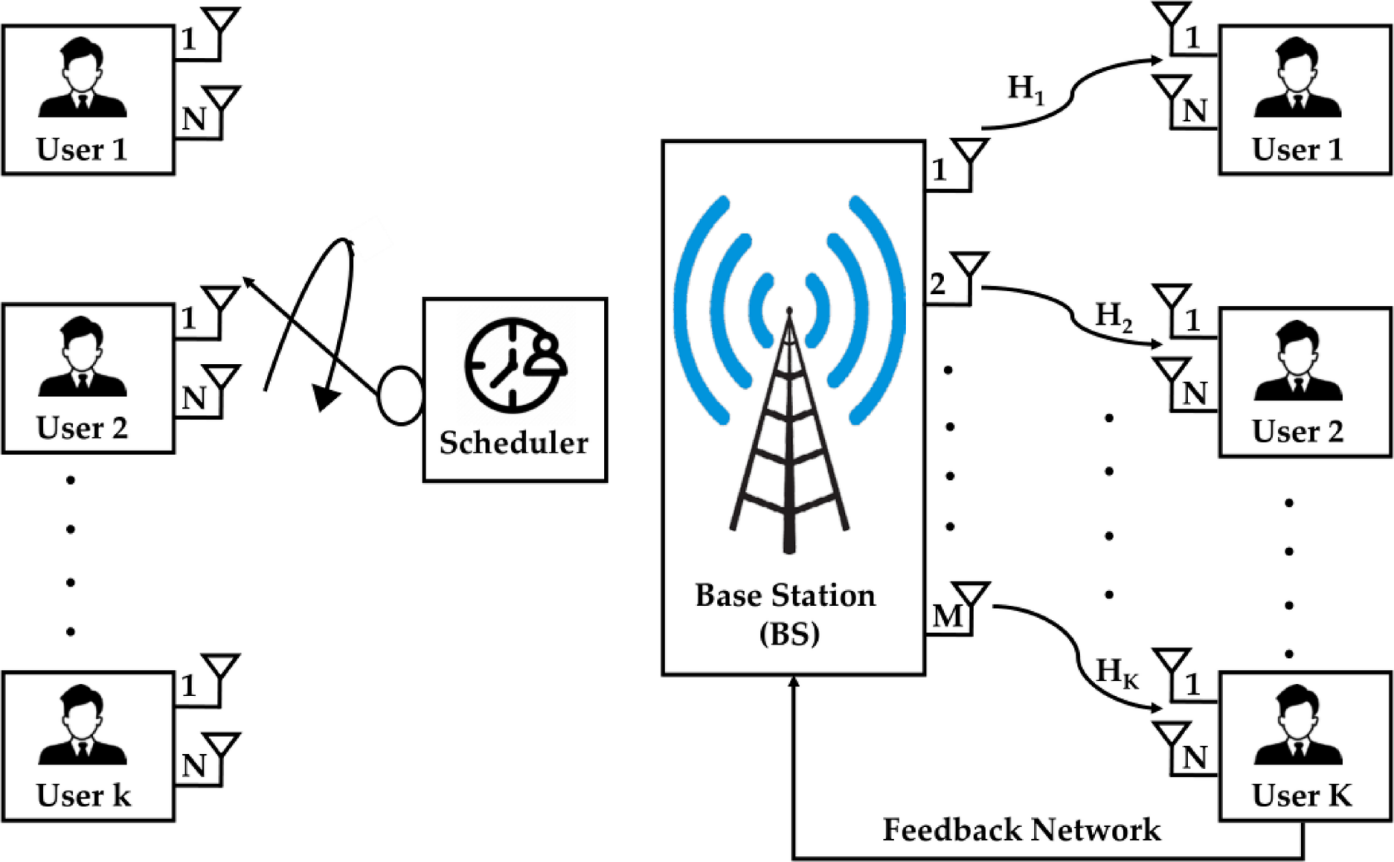
MU-MIMO architecture downlink model with M transmission antennas with K users.

The following section describes the MU-MIMO networks consisting of a base station (BS) with M number of broadcasting antennas. We have the K subscribers’ units on the receiving end of the user’s terminals. The user terminal consists of N units of receive antennas. The MIMO model considers the following constraints M > 1, N > 1, M ≥ N and K >  > M. The MU-MIMO architecture is illustrated in Fig. 1. Table 1 displays the mathematical notations, and its description used in this paper and parameters of proposed MU-MIMO antenna systems.

**Table 1:** Mathematical notations and its description.

Notation	Description
M	Total number of transmitting antennas
K	Total number of users/subscribers
N	Total number of receiving antennas
$\mathop Y\nolimits_{k}^{t}$	User received signal in the form of vector k
$\mathop W\nolimits_{k}^{t}$	Additive noise in the form of vector k
$\mathop H\nolimits_{k}^{t}$	Total number of channels in the form of vector k
$\Re_{k}$	Antenna attenuation factor
$X^{t}$	Base station
$w_{k} (n)$	Additive white Gaussian noise
$y_{k} (n)$	Received signal by user k
SINR	Signal to interference plus noise ratio
$\rho_{k}$	Signal to noise ratio
$F_{j}$	Food source location in the SSA
θ	A specific subset of K users
$\Theta$	All set of user sequence

The equation for user k at given slot (t) of the received antennas signal is represented as

2 $$\mathop Y\nolimits_{k}^{t} = \sqrt {\Re_{k} } H_{k}^{t} X^{t} + W_{k}^{t} ,k = 1,...,K,$$

where $\mathop Y\nolimits_{k}^{t}$ represents the user received signal in the form of vector ($k^{th}$) of time slot ($t^{th}$) with dimension of $N \times 1$. The communication signal in the form of vector ($X^{t}$) of time slot ($t$) with dimension of $M \times 1$. $\mathop W\nolimits_{k}^{t}$ is the additive noise in the form of vector with dimension ($N \times 1$) in the user $k$ having mean of zero and unit variance. A complex channel is represented as $\mathop H\nolimits_{k}^{t}$ with dimension of ($N \times M$) matrix. The gain coefficient is the attribute of channel matrix interaction between both transmission and receiving antenna of the user $k$_._ The shadowing effect and path loss affect the antenna attenuation of power which can be modelled by parameter $\sqrt {\Re_{k} }$_._

The signal communication between M set of antennas of the base station is denoted as $X^{t}$ in the given slot of ($t$) which is the dimension of $M \times 1$. The expected signal at receiver end of user $k$ of $n^{th}$ antenna is represented as:

3 $$y_{k} (n) = \sqrt {\Re_{k} } \sum\limits_{m = 1}^{M} {h_{k} (n,m)x(m) + w_{k} (n)}$$

We know that $w_{k} (n)$ is the additive white Gaussian noise in the receive antenna (n) of user $k$. Where $x(m)$ is considers as the actual signal for the $k^{th}$ user. The inference data with the constraint of $\overline{m} \ne m$ is denoted as signal of $x(\overline{m} )$.The SINR in $k^{th}$ receive antenna of $y_{k} (n)$ is represented as:

4 $$SINR_{m,n}^{k} = \frac{{\left| {h_{k} (n,m)} \right|^{2} }}{{\left\{ {\left( {\frac{M}{{\rho_{k} }}} \right) + \sum\nolimits_{{\overline{m} \ne m}} {\left| {h_{k} (n,\overline{m} )} \right|^{2} } } \right\}}}$$

where $\rho_{k} = (\Re_{k} /N_{o} )$ consider as the SNR in user k in an average. In this system model $\rho_{k} = \rho$, for all users of (K). The above Eq. 4$h_{k} (n,m)$ represents the channel gain between k received antenna at the base station. We assume that there is no cancel device for inference at the receiver user terminal. The sum rate calculation of MIMO antenna with upper limit as:

5 $$C_{sum} (H_{1} ,..,H_{K} ) \le {\rm E}\left[ {\sum\limits_{m = 1}^{M} {\log_{2} \left( {1 + \mathop {\max }\limits_{1 \le k \le K,1 \le n \le N} SINR_{m,n}^{k} } \right)} } \right]$$

where $C_{sum}$ represents the upper limit of MIMO antenna capacity and the channel vectors are denoted by $H_{1} ,..,H_{K}$_._ The sum-rate capacity and throughput of the proposed MU-MIMO systems user selection are same based on the above expression. Where E represents the expectation values of channel vectors. MIMO antenna capacity can be represented with maximum of SINR is given by:

6 $$C_{sum} (H_{1} ,..,H_{K} ) = \left[ {\sum\limits_{m = 1}^{M} {\log_{2} \left( {1 + \mathop {\max }\limits_{1 \le k \le K,1 \le n \le N} SINR_{m,n}^{k} } \right)} } \right]$$

## Scheduling using binary salp swarm algorithm

A new swarm intelligence optimisation called SSA was put forth by Mirjalili et al.^31^. Salps swarm together when navigating and hunting in waters, and SSA imitates this activity. It was demonstrated in^32^ that SSA performs noticeably better than reputable and recent metaheuristics. This is because SSA incorporates several stochastic operators, which makes it possible for this method to effectively reject local solutions in multifaceted search environments. The effectiveness of the SSA technique for both small- and large-scale issues was further demonstrated by Mirjalili et al. A trustworthy stochastic optimisation approach should be used to deal with a feature selection problem since it is a binary problem with many local solutions that changes dramatically as datasets are changed. This encouraged our MIMO optimization to present a user channel selection employing SSA to take advantage of adaptability. The stochastic nature of SSA algorithm is developed to manage a wide range of variable and local solutions. This paper proposes MIMO scheduling methods based on binary SSA. Since the native SSA is conventionally designed to handle real-time problems, certain changes to the SSA are necessary to address scheduling issues involving binary factors. This work focuses on the binary form of SSA, which is implemented for MIMO model scheduling after being changed from continuous to binary.

The swarm behaviour of sea creatures known as salps is the primary motivation for SSA. The tunicates known as salps are barrel-shaped, free-floating members of the salpidae group. When travelling and hunting in oceans and seas, salps frequently float in tandem in a formation known as a salp chain. A colony of salps is considered to migrate in this manner for more effective foraging and movement. SSA is a population-based method that begins by randomly initialising a predetermined number of salpidae, just like other swarm intelligent algorithms. Each of these people stands for a potential remedy to the particular issue. The salp swarm comprises two categories of salp. First one is a leader and second one is the followers. The leader is the initialize salp in the network that directs the followers’ movement. A two-dimensional matrix can express a swarm X of n salps. The leader salp swarm is targeting for food source F in the search area.

7 $$X_{i} = \left[ {\begin{array}{*{20}l} {x_{1}^{1} } \hfill & {x_{2}^{1} } \hfill & {x_{d}^{1} } \hfill \\ {x_{1}^{2} } \hfill & {x_{2}^{2} } \hfill & {x_{d}^{2} } \hfill \\ {x_{1}^{n} } \hfill & {x_{2}^{n} } \hfill & {x_{d}^{z} } \hfill \\ \end{array} } \right]$$

The mathematical representation of the salp swarm process is defined as follows. As per the salp swarm intelligence mechanism concept, the leader and followers represent the optimisation process. Therefore the mathematical model of a leader is described as follows:

8 $$x_{j}^{1} = \left\{ {\begin{array}{*{20}l} {F_{j} + c_{1} ((ub_{j} - lb_{j} )c_{2} + lb_{j} )} \hfill & {c_{3} \ge 0.5} \hfill \\ {F_{j} - c_{1} ((ub_{j} - lb_{j} )c_{2} + lb_{j} )} \hfill & {c_{3} < 0.5} \hfill \\ \end{array} } \right.$$

where $x_{j}^{1}$ and $F_{j}$ locate the present positions of leaders and food supply in the jth dimension. $c_{1}$ is a variable that steadily decreases throughout the duration of repetitions. The other variables $c_{2}$ and $c_{3}$ are the randomly selected from the interval of [0, 1]. The lower and upper limit of jth dimension is represented $lb_{j}$ and $ub_{j}$. The variable $c_{1}$ can be calculated using following expression.

9 $$c_{1} = 2e^{{ - \left( \frac{4l}{L} \right)^{2} }}$$

The followers salp positions are calculated as follow as:

10 $$x_{j}^{1} = \frac{1}{2}\left( {x_{j}^{i} + x_{j}^{i - 1} } \right)$$

Here $i \ge 2$ and $x_{j}^{1}$ indicates the current place of ithsalpfollowersin the jthdimension.

The conventional SSA pseudocode is described in Algorithm 1.
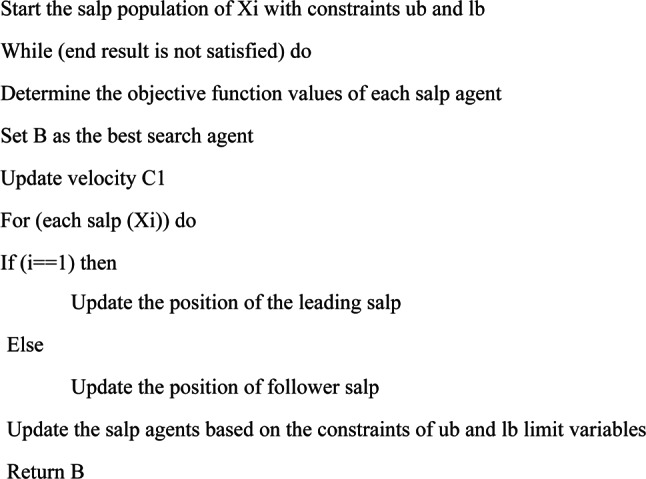


The optimisation process is started by SSA, like similar SI algorithms, by randomly creating a population of options (salps). Next, an objective function is used to assess the created solution. The fittest solution in SSA is designated as food source F, and additional solutions (follower salps) will hunt it. Each iteration updates the c1 variable with the equation of the leader’s (best salp’s) dimensions equation and the positions of the following salps with the help of the follower equation. Until a halting requirement is satisfied, the previous stages are repeated indefinitely. F should be updated during the optimisation process since the population’s solutions may be enhanced due to the exploration and exploitation operations.

### The proposed binary SSA

The SSA is a more contemporary optimizer that has yet to be used to solve MIMO scheduling issues. It has numerous distinctive qualities make it advantageous to be used as the search engine in FS and worldwide optimization issues. The SSA is initially effective, flexible, straightforward, and simple. The fact that SSA needs one parameter to balance exploitation and exploration is an added benefit. The SSA can investigate most of the search space at the beginning of the search process and then utilize the areas of greatest potential in the latter stages because this value is automatically reduced throughout iterations.

Additionally, follower salp locations change progressively concerning other swarm members, which aids the SSA in avoiding entrapment at local optima. Follower agent gradual motions can prevent the SSA from quickly degrading in local solutions. The SSA keeps the best agent thus far discovered and attributes it to the food variable, so even if the entire agent population weakens, it never disappears. The best salp developed so far, the SSA, allows the leader to travel only depending on the location of the food source, allowing the leader to continuously explore and take advantage of the area close to the food source.

Two SSA techniques are suggested in the following section using a wrapper optimization method. Since the SSA was initially created to handle continuous optimization issues, the initial step is to prepare to deal with the FS by transferring it to a binary format. Salps can move to any location in search space in the case of the continuous SSA, while they can only move between 0 and 1 values in an objective function. The follower salp locations are also updated in the initial SSA using an average operator across a solution and its neighbour. In the second method, a straightforward crossover operator takes the place of the average operator and serves the same purpose of improving SSA’s exploratory activity.

### Binary SSA (BSSA) with objective functions

Objective functions (OF) are one of the most effective strategies, according to Rizk-Allah et al.^33^, to transform a continuous algorithm into a binary one. This study converts the continuous SSA to binary form using binary theory. The MIMO systems and the channel selection function are distinct. An objective function specifies the likelihood of updating a channel subset element to be either 1 (selected) or 0 (not selected), as the following equation is developed to convert the original SSA to a binary version.

11 $$f\left( {x_{j}^{i} (t)} \right) = \frac{1}{{1 + \exp^{{ - x_{j}^{i} (t)}} }}$$

where $x_{j}^{i}$ defines the ith channel in the dimension of jth and t denotes the present iteration.

In MIMO system, a channel of selection in the next iteration is updated as follow as:

12 $$x_{j}^{k} (t + 1) = \left\{ {\begin{array}{*{20}l} 0 \hfill & {If\,rand\, < \,f(v_{i}^{k} (t + 1))} \hfill \\ 1 \hfill & {If\,rand\, \ge f(v_{i}^{k} (t + 1))} \hfill \\ \end{array} } \right.$$

where $x_{j}^{k} (t + 1)$ is the kth channel at jth dimension in f result, $f\left( {x_{j}^{i} (t)} \right)$ used to calculate the probability value, which can be applied to decide binary value 0 and 1. Since the binary salp swarm optimization is suitable for channel selection in the multiuser MIMO antenna systems. The following section provide the detailed steps for DPC and user scheduling model using BSSA.

## DPC scheduling using binary SSA

The scheduler must choose the optimal M receiving antennas from an aggregate of N, K receiving antennas to pre-cancel known interference at the transmitter where the BS may sustain M users using the strong information-theoretical result provided by DPC. For the DPC, the order in which users encode data is crucial. Equation (1) can be used to determine the Selection of M users with different encoding orders. However, the quantity is too great to be considered at millisecond periods. With some difficulty managed by the algorithm that the BS will analyse, the BS can handle complicated arithmetic operation that involve signal processing. We assess a portion of user sequences while adhering to the following restrictions:

1. Only one evaluation is required for a pair of identical M users.
2. We think that the wide bandwidth selective channel can connect the BS. Diverse users can constantly have a user sequence without repetition.
3. At never time should an antenna that transmits be inactive. By allocating M users to M broadcasting antennas, this can be accomplished.

By using such restrictions, the number of possible scheduling scenarios is reduced below that of ESA (DPC) and is represented by

13 $$N_{Unique\_user\_sequence} = = \left( {\begin{array}{*{20}c} K \\ M \\ \end{array} } \right)$$

With a spike in users and sending antennas, the amount may rise rapidly, adding complexity. This article discusses a less computationally demanding binary salp swarm algorithm for locating suboptimal solutions. The term θ refers to a specific subset of M users among K users. Consider $\theta \in \Theta$ (which includes all set of user sequence) and $\theta_{i} = \left\{ {\theta_{i}^{1} ,\theta_{i}^{2} ,\theta_{i}^{3} ..,\theta_{i}^{M} } \right\},\,1 \le i \le \left| \Theta \right|$. At the present, MIMO model selection obtained through a sequence of M users (θ) is calculated by:

14 $$C_{sum} (\theta ,H_{1} ,..,H_{K} ) = \left[ {\sum\limits_{m = 1}^{M} {\log_{2} \left( {1 + \mathop {\max }\limits_{k \in \theta ,1 \le n \le N} SINR_{m,n}^{k} } \right)} } \right]$$

The number of user antennas in the present user set (θ) are kept as $N_{i} = \left\{ {n_{i}^{1} ,n_{i}^{2} ,n_{i}^{3} ..,n_{i}^{M} } \right\}$. This set is determined by the BS antenna with the highest SINR. Scheduling reception antennas with broadcasting antennas is a contemporary sequential optimisation problem, that is,

15 $$\max_{\theta \in \Theta } \sum\limits_{m = 1}^{M} {\log_{2} \left( {1 + \mathop {\max }\limits_{1 \le k \le K,1 \le n \le N} SINR_{m,n}^{k} } \right)}$$

The user selection process is optimized using following expression as $\theta_{opt} = \arg \max_{\theta \in \Theta } C_{sum} \left( {\theta ,H_{1} ,H_{2} ,..,H_{k} } \right)$. The sequence of user antennas obtained for the group of users in $\theta_{opt}$ is calculated as: $N_{opt} = \left\{ {N_{{\theta_{opt}^{i = 1} }}^{i = 1} ,N_{{\theta_{opt}^{i = 2} }}^{i = 2} ,..,N_{{\theta_{opt}^{i = M} }}^{i = M} } \right\}$, and the value of $N_{{\theta_{opt}^{i} }}^{i} = \arg max_{1 \le n \le N} SINR_{i,n}^{{\left( {\theta_{opt}^{i} } \right)}}$. ESA assesses each potential combination in order to maximise. Even if scheduling is ideal, it must be finished in a matter of milliseconds. Due to this, ESA is operationally costly and inefficient when compared to the scheduling needs. As it mimics salp swarm behaviour, binary SSA has reduced computational demands and achieves near-optimal performance at much faster rates. It is thus appropriate for scheduling issues.

## Binary FPA for user scheduling

The following list of variables and motions used in binary SSA:

- Z is used to refer to the utility coefficient shown in Eq. (9).
- The population size is indicated by Pop_size.
- P stands for proximity probability in the range [0, 1].
- D contains a binary representation of the scheduled users that is M [log_2_K] in length.
- The step size parameter, L, is derived from the Levy distributions.
- The repetition number is Max_itr.
- The M users that will be served and are stored in SelectedUT are a M size row vector and were chosen by BS.
- Selectedreceivedantenna, which holds the receive antennas chosen by the picked UTs present in SelectedUT, remains a size M vector.

Step 0:

1. Initialise the population with binary information once it has been generated at random in D-dimensional searching spaces.
2. In the proposed binary SSA, Eq. (12) generates the initial solutions.
3. Each row is represented by a set of objects ($\theta$), each of which is a binary string ($\mu$).
4. The $m^{th} \log_{2} K$ bits of $\mu$ include the binary representation of the user who was randomly chosen to benefit from the $m^{th}$ antenna for transmission.
5. As in the scenario when K = 15, N = 3, and M = 4. Let’s say that four BS broadcast antennas are assigned to serve the third user, the sixth user, the 12th user, and the 13th user at random. Therefore, [log_2_15] = 4 is the number of bits needed for each user. If so, [0, 0, 1, 1] will be the user sequence ($\theta$) corresponding to these collections.

Step 1:

The following set of restrictions is provided for improved binary SSA efficiency:

1. There should not be any duplicate user indices in any of the population’s rows since the transmitting antenna must deliver separate users’ different streams of data.
2. Each population row should be similar, allowing for quicker testing of various user settings.
3. No user index shall be zero or K units higher than the total number of users.

If any single gamete violates any restriction, then specific bits must be arbitrarily toggled. This procedure can be repeated until none of the restrictions are violated.

Step 2:

1. Calculate $\Theta$ using the current population value.
2. By evaluating Eq. (9), update the fitness function of each salp.
3. The salps are arranged in decreasing order of fitness function.
4. Update $$selectedantenna = N_{opt}$$ and $$selectedusers = \theta_{opt}$$.

Step 3:

1. It is assumed throughout this work that the value of $p$ is 0.8.
2. Using Eq. (11), determine a D-dimensional step vector L for every flower in the population that follows a Levy distribution for each iteration and dimension.
3. Using Eq. (10), update the overall optimum position.

Step 4:

1. Finding random salps in the vicinity and updating the optimal location based on Eq. (12) if $count < \max \_itr$ and random value $(rand) < p$. Retrace your steps starting at Step 2 and finish.
2. Add one more to the total.
3. Assess the results ($\Theta$) using the new population.
4. The primary goal of fitness, which is determined by Eq. (9), will be the system’s throughput.
5. Update $$selectedantenna = N_{opt}$$ and $$selectedusers = \theta_{opt}$$.
6. Determine the user, best user, and best receiving antenna indices using the information provided above.

**Fig. 2:**
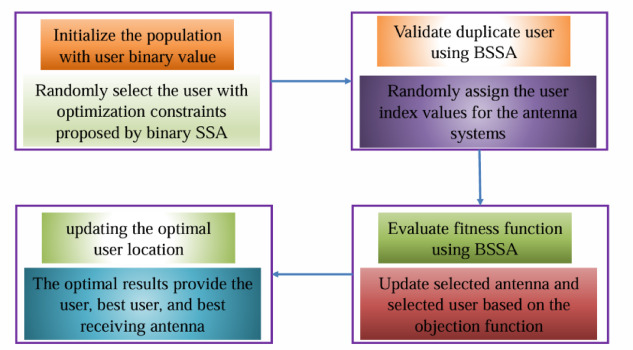
The proposed binary SSA based user selection optimization model in MU-MIMO system.

The state diagram of the proposed binary SSA based MU-MIMO systems user selection model is illustrated in Fig. 2. The step-by-step flow for the user selection in MIMO systems explained in the given Fig. 2.

## Experimental results and discussions

This section compares the efficacy of binary SSA, binary BA^34^, binary GA, binary FPA^35^, ESA (DPC) and other state of art optimization techniques while also analysing the simulation results that are obtained under various scenarios. To highlight the advantages of these techniques, we have offered an analysis of the network’s sum rate for binary SSA, binary FPA, binary BA, binary GA, PSO, SSA, FPA and ESA. The binary SSA surpasses the binary BA, binary GA, binary FPA, PSO, SSA, FPA and random search method in determining higher feasible system sum-rate/throughput for the MU MIMO downlink antenna systems, according to the simulation findings reported in this section. This section also presents and discusses binary SSA meta-heuristic outcomes. The findings from the simulation, demonstrating that the feasible structure sum rate of various MU MIMO BC systems increases with a rise in the population size (i.e. Pop_size) and the total amount of generations/iterations (i.e. Max_itr), are provided below to demonstrate the meta-heuristic the real world of the binary SSA. These outcomes are in line with the meta-heuristic methodology.

This section also discusses and presents the deviations from the ESA (DPC) produced by various methods such as binary GA, binary BA, binary FPA, and binary SSA for various system circumstances. To demonstrate how well these three meta-heuristic algorithms/approaches may converge in a MU MIMO broadcast situation, the per-generation performance of binary SSA, binary FPA, binary BA, and binary GA is also examined. The convergence performance of binary SSA is superior to that of binary BA and binary GA, as demonstrated in this section’s later portion. Additionally, the next section has completed the complexity analysis for several optimization techniques. This complexity analysis illustrates the benefits of binary SSA over ESA in terms of temporal and computing complexity. Additionally, the computational complexity has been examined concerning how often the objective/cost function has been evaluated, as given in Eq. (9).

**Fig. 3:**
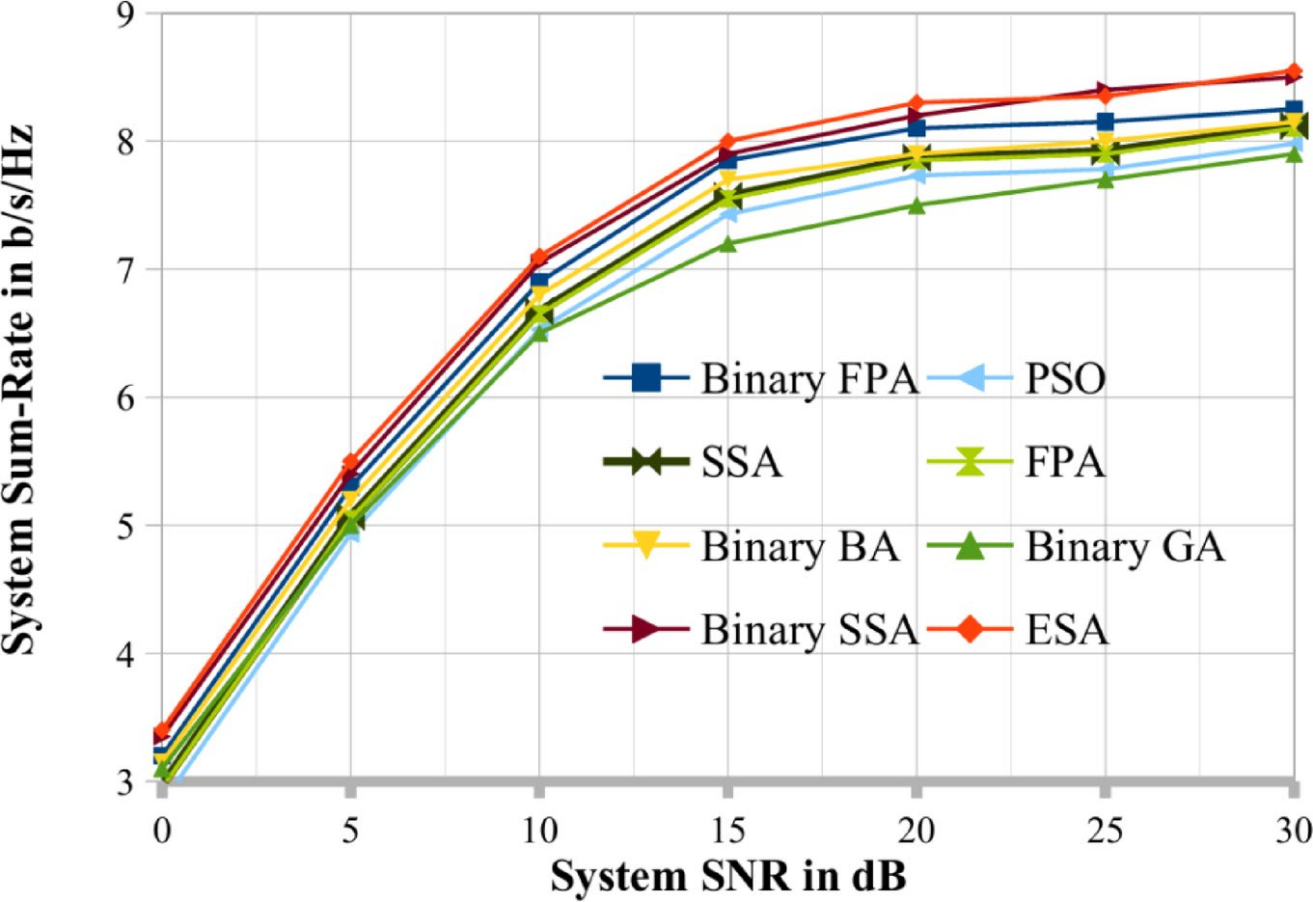
The proposed system sum rate compared with existing optimization techniques when K = 20.

**Fig. 4:**
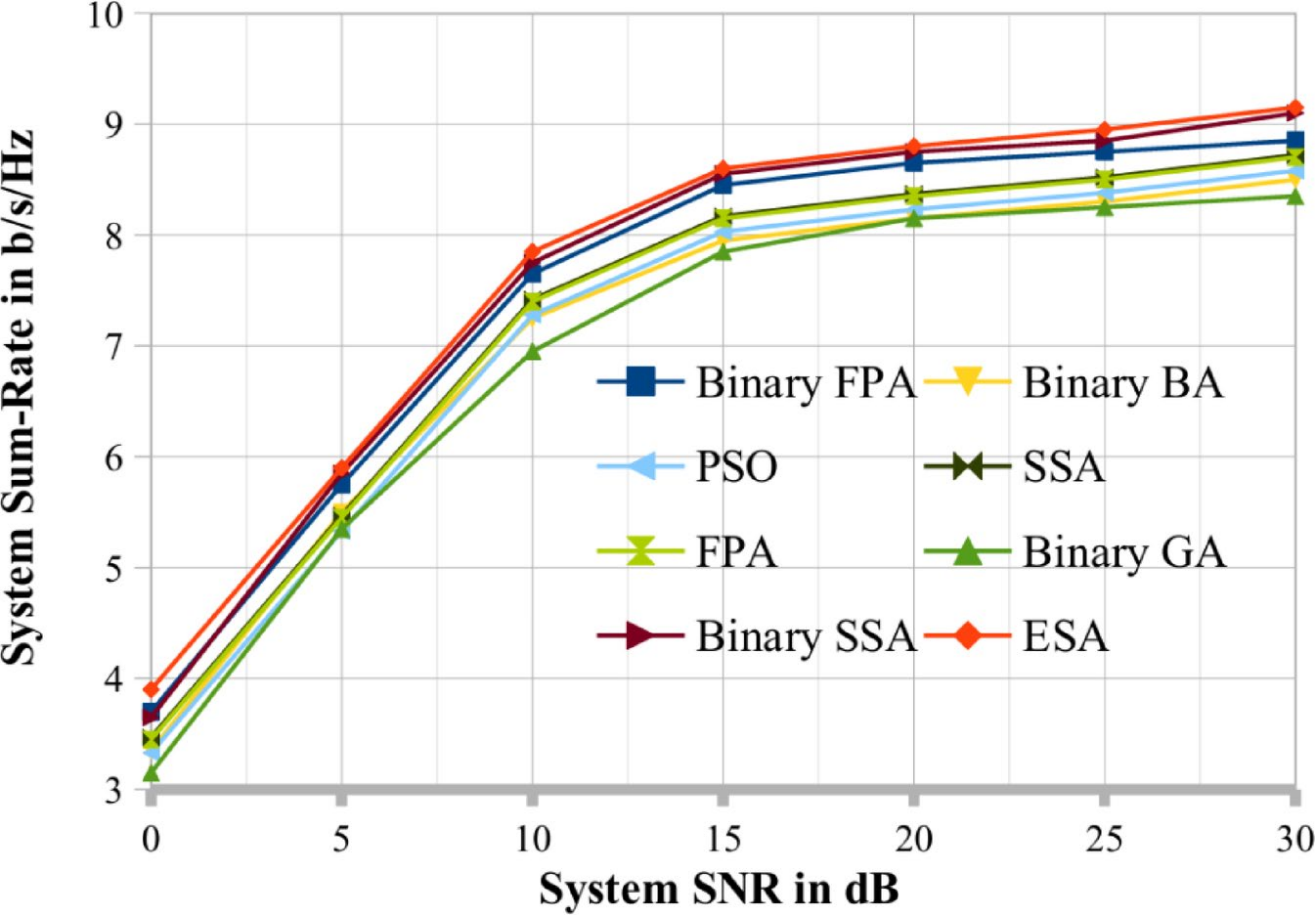
The proposed system sum rate compared with existing optimization techniques when K = 25.

**Fig. 5:**
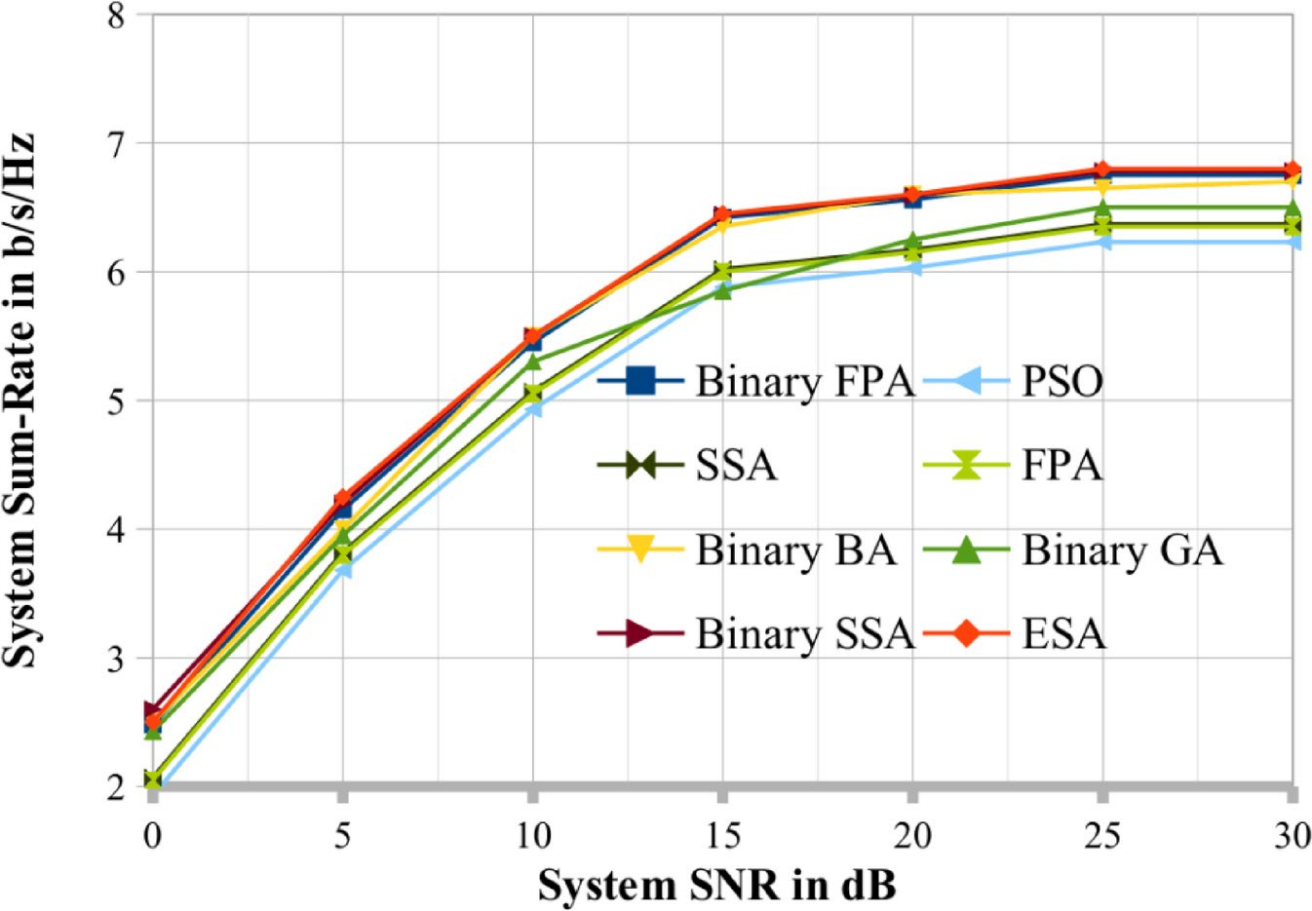
The proposed system sum rate compared with existing optimization techniques when K = 10.

To compare the results, we choose two cases, respectively shown in Figs. 3 and 4, where (K, N, M, Pop_size, Max_itr) = (20, 4, 6, 20, 30) and (25, 5, 7, 25, 30). A random search procedure selects the user series at random. According to the graphs, binary SSA performs better and close to optimally than binary BA, binary GA, PSO, SSA, FPA and binary FPA methods. This study set the binary GA’s crossover and mutation probabilities at 1.0 and 0.1, respectively^30^. Therefore, for the remaining results reported in this study, the results obtained by the binary SSA method are thoroughly contrasted with those of ESA (DPC). Figures 4 and 5 make it abundantly evident that the binary SSA outperforms binary BA, binary FPA and binary GA regarding system throughput for the same number of Pop_size and Max_itr. This demonstrates how the binary SSA outperforms the binary BA and binary GA as a searching algorithm. The binary BA performs better than the binary GA in achieving a greater systems sum rate. Therefore, the binary SSA is a superior meta-heuristic method to the binary BA and binary GA for the scheduling process of MU-MIMO broadcast scenarios.

For a large range of MIMO SNR values, it is determined that binary SSA achieves optimal system capacity as a DPC. Each of these estimations represents the average of 1000 different iterations. Each run channel matrix for each user (H1,…, Hk) is generated independently.

For the two circumstances listed below, the binary SSA’s meta-heuristic nature is being assessed as follows:

- For decreasing the size of the population (Pop_size)
- The number of generations decreases (Max_itr).

**Fig. 6:**
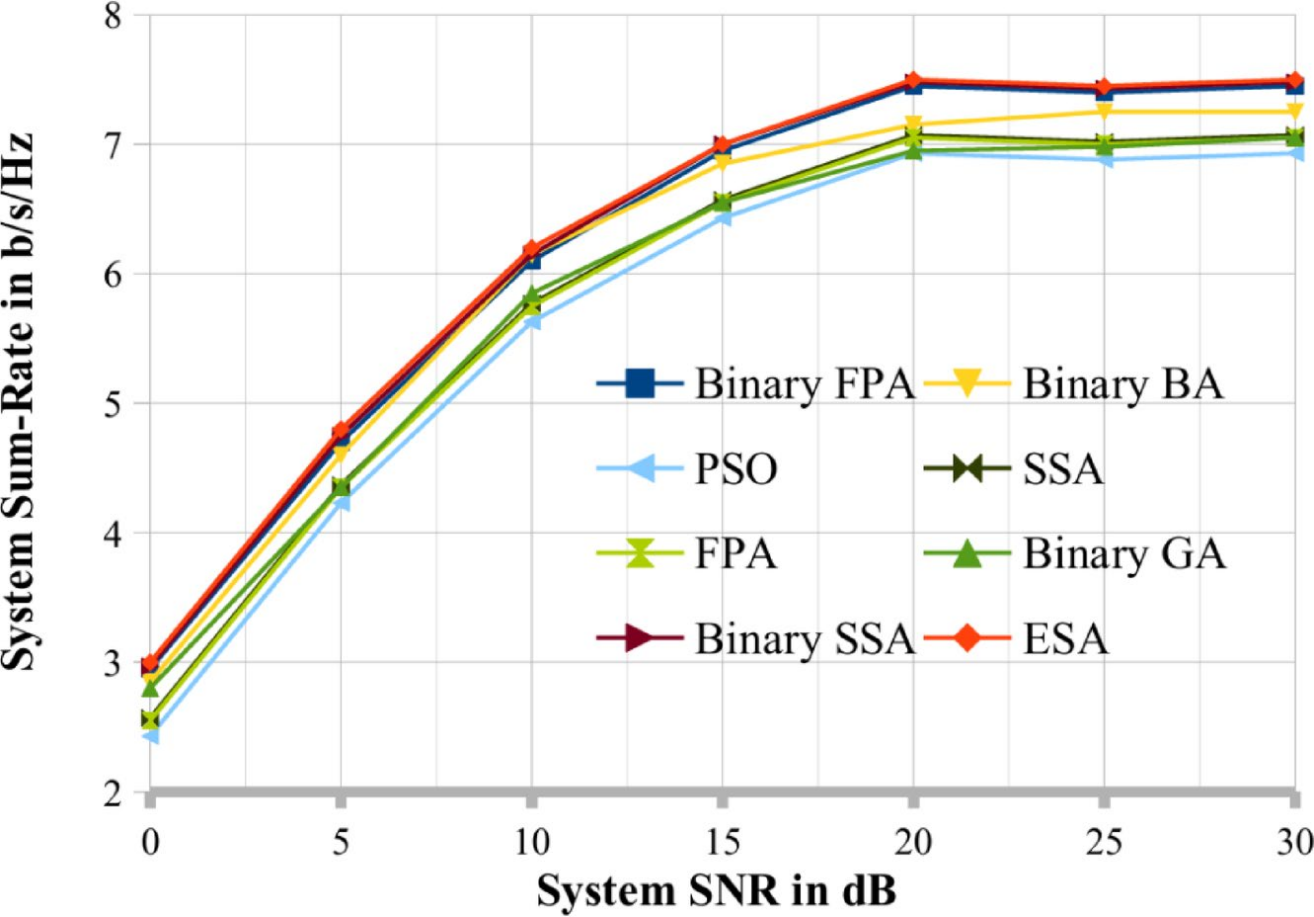
The proposed system sum rate compared with existing optimization techniques when K = 12.

**Fig. 7:**
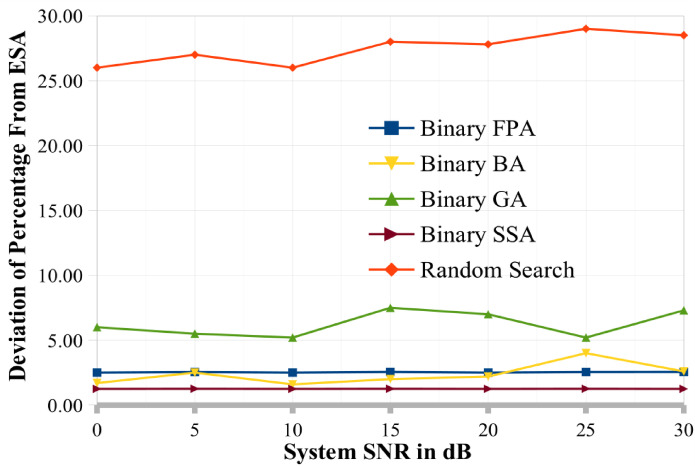
Percentage deviation of the proposed and existing optimization techniques from ESA model when K = 10.

Figures 4 and 5 show the performance of the binary SSA and the system sum rate for various values of the SNR. In addition, K = 10, N = 2, M = 4, Max_itr = 10, and Pop_size = 30 are considered. There have been three population sizes observed. Figure 5 shows that when population size decreases, the system sum-rate capacity of the MU MIMO broadcast system generated via binary SSA also decreases. This is in line with the properties of the meta-heuristic. Like Figs. 5, 6 evaluates the binary SSA’s performance as the number of generations/iterations (Max_itr) increases. Figure 6 considers the following system parameters: K = 12, N = 3, M = 5, Pop_size = 12, and Max_itr = 30. There have been three generation numbers examined. As demonstrated in Fig. 7, the binary SSA’s system sum-rate capacity for the MU MIMO broadcast system grows as the generation/iteration numbers are reduced. Figure 6's depiction of this observation and behaviour likewise complies with the traits of meta-heuristic algorithms. The population sizes and iteration sizes conform that the proposed BSSA optimization techniques mostly suitable for large scale MU-MIMO systems. Also in the same number of iteration and population sizes the existing optimization lacks its performance in terms of sum rate.

The ESA (DPC) result is the best value for calculating the output variable using various methodologies^25^. For a random search, binary BA, and binary FPA, we calculated the percentage deviation from ESA (PDESA). The formula for the percentage divergence from ESA is:

16 $$PDESA(\phi ) = \left( {\frac{{\left( {C_{sum}^{ESA} - C_{sum}^{\phi } } \right)}}{{C_{sum}^{ESA} }} \times 100} \right)$$

**Fig. 8:**
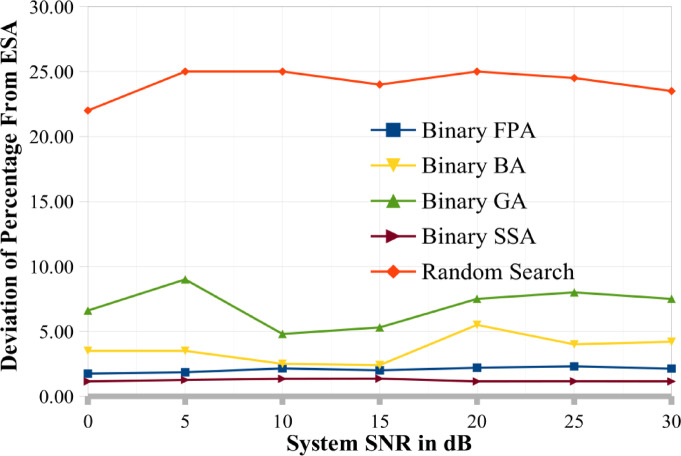
Percentage deviation of the proposed and existing optimization techniques from ESA model when K = 12.

Figures 7 and 8 show the PDESA for two different situations. It is reasonable to assume that binary SSA is superior to random search, binary BA, binary GA, and binary FPA across a wide range of system SNR values since it has the lowest PDESA (close to 0). The PDESA values for the random search approach are very high. The estimate of 1000 independent runs are also included for each point in these graphs. These numbers show that the PDESA achieved by binary SSA is in the range of 0% to 1%, the PDESA achieved by binary FPA, binary BA and binary GA is in the range of 2–5%, and the PDESA achieved by binary SSA is in the range of 4% to 9%, correspondingly. The random search technique produced a PDESA between 21 and 30%. Therefore, the PDESA achieved by binary SSA is near 0%, preferred for MIMO systems among all alternative techniques.

Thus, regarding per-generation efficiency for MU MIMO broadcasting situations, the binary FPA has a bigger advantage over the binary BA and binary GA. In terms of per-generation and resolution efficiency, binary BA outperforms binary GA. For the performance comparison, Tables 2 and 3 additionally include a tabular representation of the generation-wise effectiveness of the binary SSA, binary FPA, binary BA, and binary GA. We can see from both tables that binary SSA increases the feasible system sum rate at a faster pace than binary FPA, binary BA and binary GA. Furthermore, binary BA increases the possible system sum rate faster than binary GA. Additionally, the binary GA’s growth rate in the feasible system sum rate is the slowest. Therefore, additional generations or a higher population size are needed for the binary GA to obtain the ideal system sum-rate value. The binary SSA method is superior to the binary BA and binary GA approaches.

**Table 2:** Sum rate performance comparison when K = 20.

Generation number	Binary SSA (b\s\Hz)	Binary FPA (b\s\Hz)	Binary BA (b\s\Hz)	Binary GA (b\s\Hz)
1st	10.8862	10.6762	10.4862	10.4688
2nd	10.8972	10.6772	10.4972	10.4688
3rd	10.8986	10.7289	10.4986	10.5211
4th	10.9268	10.7298	10.5268	10.5268
5th	10.9278	10.8278	10.5778	10.5478
6th	10.9488	10.8248	10.6478	10.5488
7th	10.9662	10.8266	10.6662	10.5562
8th	11.0245	10.8402	10.7245	10.5645
9th	11.1254	10.8412	10.7454	10.5654
10th	11.2435	10.8454	10.7535	10.5635

**Table 3:** Sum rate performance comparison when K = 25.

Generation number	Binary SSA (b\s\Hz)	Binary FPA (b\s\Hz)	Binary BA (b\s\Hz)	Binary GA (b\s\Hz)
1st	12.6852	11.6862	11.4662	11.4658
2nd	12.6962	11.6972	11.4772	11.4678
3rd	12.7976	11.7689	11.4986	11.5291
4th	12.8288	11.7898	11.5068	11.5263
5th	12.8298	11.8578	11.5378	11.5478
6th	13.1408	11.8848	11.6078	11.5498
7th	13.1692	12.1266	11.6462	11.5502
8th	13.2285	12.1402	12.7245	11.5645
9th	13.4274	12.2412	12.7754	11.5664
10th	13.4465	12.2454	12.7835	11.5695

## Complexity analysis

Binary SSA and DPC’s computational challenges are compared in this section. The computation required is determined by how frequently the utility function is computed. As described in^36^, an indicator of computational complexity is defined as the sum of arithmetic operation performed by the different optimization techniques. From Eq. (4), 2 M CAMs are necessary to calculate SINR. The user has a total of N*M SINR terms.

Therefore, 2M2N CAMs are needed for Eq. (6) for a single user. Each M-user sequence Thus, ESA needs a certain quantity of CAM. Binary SSA calls for 2M3N × Pop_size × Max_itr number of CAM. Contemporary data interaction uses specialised multicore DSP processors, such as Texas Instruments DSP processor 66AK2Ex, to perform simulation experiment. Table 4 includes a discussion of this. We can infer from the table that binary SSA requires significantly less processing time than ESA. However, as shown in earlier graphs and plots, the possible system sum-rate capacity performance of binary SSA and ESA (DPC) is nearly the same. Binary SSA and binary BA have almost the same period. Binary FPA exhibits much greater feasible system sum-rate capacity efficiency than binary BA.

**Table 4:** Computational complexity of ESA (DPC) and binary SSA comparisons for MU-MIMO system.

Design parameters (Max_itr = 30)	ESA (DPC)		Binary SSA	
[K,N,M, Pop_size]	CAM	Time (ms)	CAM	Time (ms)
[12,2,4, 12]	97900	0.3593	57360	0.0371
[17,3,5, 20]	3272260	2.6118	425800	0.0740
[25,4,6, 22]	21739000	58.7844	1520470	0.2980
[27,5,7, 25]	26498020000	1217.4825	2992700	0.3545

Table 4 also illustrates the time complexity of both algorithms, the binary SSA and ESA (DPC). It will take more than one coherence period to complete the number of CAMs that the ESA (DPC) requires for a greater number of users. However, it takes a fraction of a second to complete the number of CAMs that binary SSA requires for larger users. This is close to the coherence time frame. The channel’s characteristics between the BS transmit antennas, and the receive antennas of all users are presumed to be block fading and quasistatic, making this observation essential for wireless communication applications. The CSI is only valid under this supposition for a brief period. The system’s CSI changes after a short while. As a result, since the fading coefficients are supposed to be constant, the user and antenna scheduling completed for one block time will not apply to the subsequent block period. Because the binary SSA has a far lower temporal complexity than the ESA (DPC), it is preferred over the latter.

## Convergence analysis

**Fig. 9:**
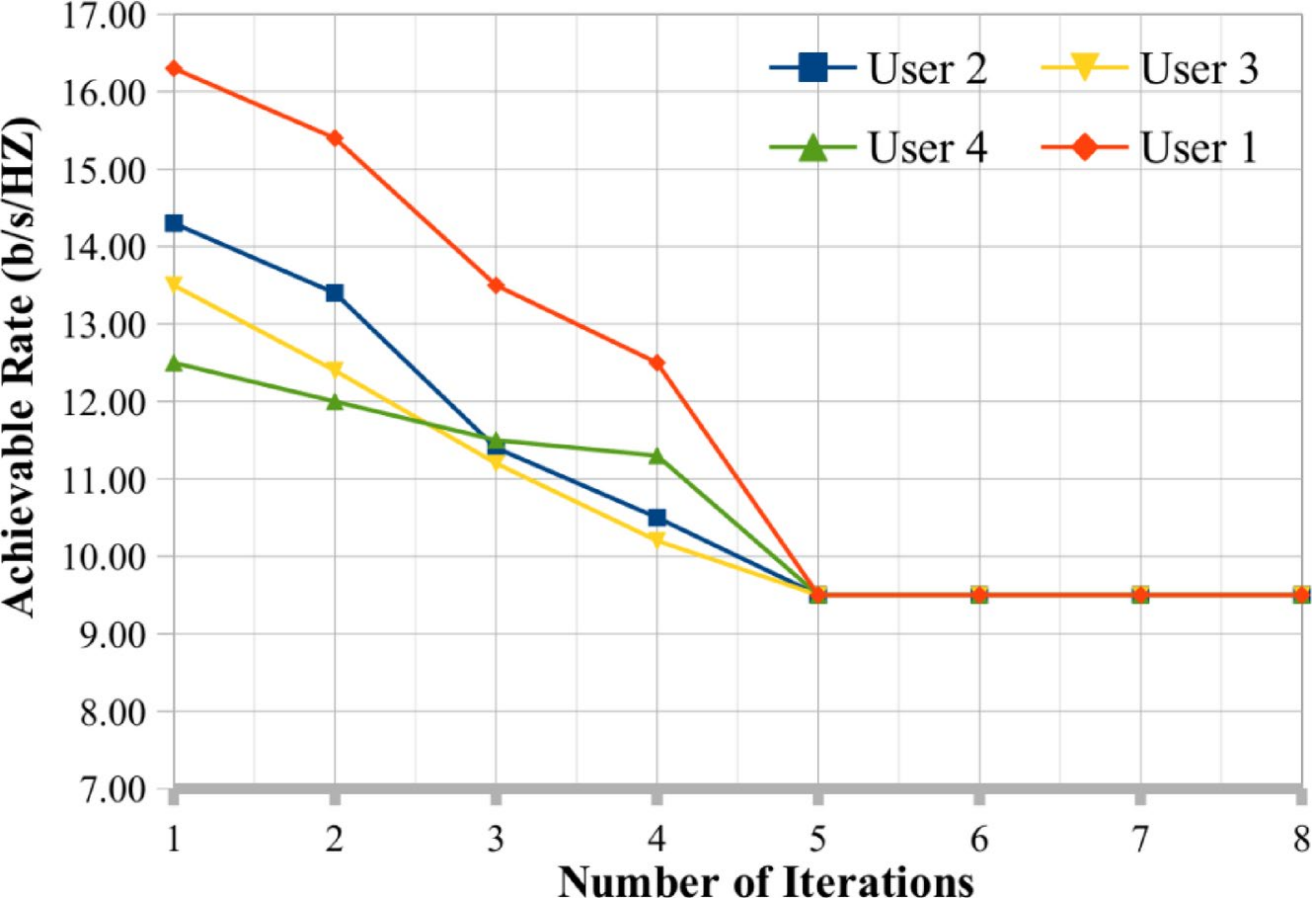
Convergence analysis of proposed technique user selection SNR rate when K = 10.

**Fig. 10:**
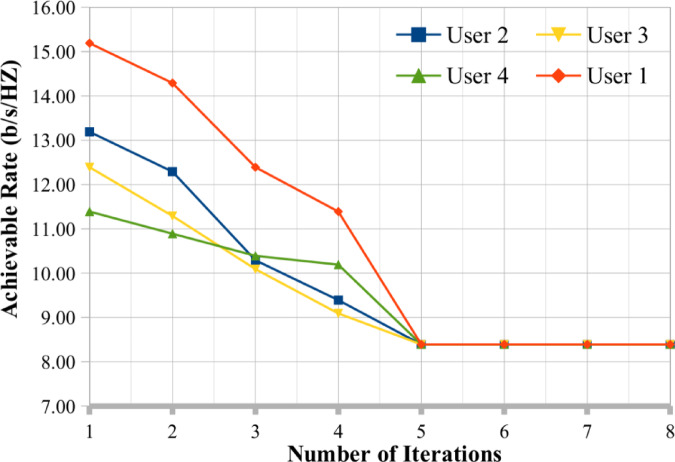
Convergence analysis of proposed technique user selection SNR rate when K = 12.

The proposed hybrid algorithm for MIMO antenna user selection optimization problem convergence analysis discussed in this subsection. Figures 9 and 10 presents the convergence analysis for different user selection based on the MIMO antenna and we have fixed our K value as 10 and 12 respectively. Based on the analysis we have observed that the proposed optimization algorithm provides the better convergence rate for user selection for MIMO antenna systems. Figures 9 and 10 show the convergence analysis of proposed optimization techniques when number of users is 4 and SNR 20 dB. In Fig. 9, the value of K = 10 and SNR is 20 dB, due to algorithm nature of factorized parameters, the hybrid optimization techniques provide the better results in a smaller number of iterations. Because the convergence speed depends on the SSA optimization characteristics of the proposed algorithm. In Fig. 10, whereas the K value is changed to 12 and we keep the SNR values is same as previous one for convergence analysis. The proposed hybrid algorithms rapidly converge to the SNR rate and minimizes the number of iterations. We observer that, the convergence rate of the proposed optimization techniques better for user selection in multiuser MIMO antenna systems.

## Conclusion

The application of binary SSA for effective antenna and user scheduling for MU MIMO broadcast channels has been examined in this study. Binary SSA has been discovered to attain a significantly greater sum rate with a significantly lower time and computational complexity than ESA. Additionally, the binary SSA demonstrated noticeably higher system throughput than the other two meta-heuristic schemes compared to binary BA and binary GA. In addition, the suggested binary SSA technique achieves higher system throughput than some competing suboptimal scheduling algorithms currently in use. The experimental results shows that the traditional user selection techniques become impractical when the number of users and antennas rises because the search space grows exponentially. In Massive MIMO settings, handling many users requires proposed binary optimization algorithms. Simulation results support these findings. Compared to binary BA, binary GA, binary FPA, and the random search scheduling algorithms from the literature, the suggested binary SSA algorithm achieves a solution that is extremely close to the optimal value rather quickly and well inside the coherence time for modern wireless data communications. For the binary SSA, there are far fewer evaluations and computations of the objective and cost functions than there are for the ESA. The binary SSA has a much lower computational cost and time complexity than the ESA. The suggested binary SSA technique also performs well generation-wise compared to binary BA, binary FPA and binary GA. Additionally; the binary SSA illustrates how meta-heuristic algorithms behave. Binary SSA may be a potential contender for implementing effective user and antenna scheduling in MU MIMO broadcast scenarios, according to the various findings of this work. In future work, heuristic-based methods are challenging to apply in actual MU MIMO systems, yet they perform well in simulations. The algorithm’s real-time flexibility, hardware limitations, and latency constraints must be considered.

## Data Availability

The datasets used and/or analysed during the current study available from the corresponding author on reasonable request.
